# Complex Virome in a Mesenteric Lymph Node from a Californian Sea Lion (*Zalophus californianus)* with Polyserositis and Steatitis

**DOI:** 10.3390/v12080793

**Published:** 2020-07-23

**Authors:** Eda Altan, Martha A. Delaney, Kathleen M. Colegrove, Terry R. Spraker, Elizabeth A. Wheeler, Xutao Deng, Yanpeng Li, Frances M. D. Gulland, Eric Delwart

**Affiliations:** 1 Vitalant Research Institute, 270 Masonic Ave, San Francisco, CA 94118, USA; ealtan@vitalant.org (E.A.); Xdeng@vitalant.org (X.D.); Yli@vitalant.org (Y.L.); 2 Department of Laboratory Medicine, University of California San Francisco, San Francisco, CA 94118, USA; 3 Zoological Pathology Program, College of Veterinary Medicine, University of Illinois at Urbana-Champaign, 3300 Golf Road, Brookfield, IL 60513, USA; delane10@illinois.edu (M.A.D.); kcolegro@illinois.edu (K.M.C.); 4 Veterinary Diagnostic Laboratory, College of Veterinary Medicine and Biomedical Sciences, Colorado State University, 300 West Drake Road, Fort Collins, CO 80526, USA; terry.spraker@colostate.edu; 5 Department of Veterinary Microbiology and Pathology, College of Veterinary Medicine, Washington State University, Bustad 471, Pullman, WA 99164, USA; elizabeth.a.wheeler@wsu.edu; 6 Karen C. Drayer Wildlife Heath Center, School of Veterinary Medicine, University of California at Davis, One Shields Avenue, Davis, CA 95616, USA; fmdgulland@ucdavis.edu

**Keywords:** California sea lion, *protoparvovirus*, polyomavirus, virome, next-generation sequencing, granulomatous, perivascular, polyserositis, steatitis, *Zalophus californianus*, vasculitis

## Abstract

An emaciated subadult free-ranging California sea lion (Csl or *Zalophus californianus)* died following stranding with lesions similar to 11 other stranded animals characterized by chronic disseminated granulomatous inflammation with necrotizing steatitis and vasculitis, involving visceral adipose tissues in the thoracic and peritoneal cavities. Histologically, affected tissues had extensive accumulations of macrophages with perivascular lymphocytes, plasma cells, and fewer neutrophils. Using viral metagenomics on a mesenteric lymph node six mammalian viruses were identified consisting of novel parvovirus, polyomavirus, rotavirus, anellovirus, and previously described Csl adenovirus 1 and Csl bocavirus 4. The causal or contributory role of these viruses to the gross and histologic lesions of this sea lion remains to be determined.

## 1. Introduction

California sea lions (Csl or *Zalophus californianus*) are considered sentinels of ocean health due to their long lifespans, feeding at a high trophic level, and sharing a coastal habitat with humans [1,2]. Wildlife rehabilitation centers, such as The Marine Mammal Center (TMMC) in Sausalito, California, provide critical care to this marine species and afford opportunities to acquire valuable biological, clinical, and postmortem data, vital to understanding environmental pressures on sea lion populations. 

Here we describe an unusual disease syndrome without a confirmed etiology found in a subadult California sea lion and our initial attempt to identify a viral cause for this condition. Similar lesions were found in eleven additional juvenile and subadult sea lions stranding over the course of several months that same year, but that were not observed for 10 previous or consequent years. The perivascular distribution, organs affected, and character of the inflammation were reminiscent of non-effusive (dry) feline infectious peritonitis (FIP), caused by the mutated form of feline enteric coronavirus (FECV), and a similar disease in ferrets [3,4]. As part of a diagnostic evaluation to screen for a potential viral cause, a mesenteric lymph node sample was analyzed using viral metagenomics to identify viral sequences. Nearly complete genomes of a novel parvovirus and polyomavirus and partial genomes of another four viruses (an adenovirus, rotavirus, anellovirus, and bocavirus) could be generated from this single mesenteric lymph node. Custom probes targeting the novel parvovirus and polyomavirus failed to detect viral RNA in histologic sections using in situ hybridization. To date, the cause of this suite of lesions has yet to be identified; however, an infectious etiology, specifically a viral pathogen, is suspected. 

## 2. Materials and Methods

### 2.1. Necropsy

An approximately 2 to 3 years old (subadult) female free-ranging California sea lion (“Hanchett”) stranded in San Luis Obispo, CA, USA, and was transported to The Marine Mammal Center (TMMC) in Sausalito, CA, USA, in June 2010 in poor body condition and subsequently died. Prior to death, blood was collected via standard procedures and hematologic and serum biochemical analyses were completed. Gross examination was performed within 48 h of death. Tissues from major organs were collected at necropsy, fixed in 10% neutral-buffered formalin, routinely processed, paraffin embedded, sectioned at 5 µm, and stained with hematoxylin and eosin (HE) for histologic evaluation. Animal necropsy was performed under MMPA permit number 18786 to F.M.D.G. Select tissues were frozen at −80 °C.

### 2.2. Protein Electrophoresis, Serology, and Immunohistochemistry

Serum was sent to Colorado State University Veterinary Diagnostic Laboratory (Fort Collins, CO) for protein electrophoresis [5]. Sera were also submitted to California Animal Health & Food Safety Laboratory (CAHFS, Davis, CA) for *Leptospira* spp. antibody titer analysis (MAT) according to previously described methods [6]. This analysis includes the following serotypes: Bratislava, Canicola, Grippotyphosa, Hardjo, Icterohemorrhagiae, and Pomona.

Representative sections were immunostained with a monoclonal antibody targeting the nucleocapsid of feline enteric coronavirus with species cross reactivity to canine, ferret, and SARS coronaviruses, according to manufacturer’s instructions (FIPV3-70, Custom Monoclonals International, West Sacramento, CA) and counterstained with hematoxylin. 

### 2.3. Viral Metagenomics

A frozen mesenteric lymph node tissue sample was homogenized with a hand-held rotor in approximately 10× volume of phosphate-buffered saline and then rapidly frozen and thawed on dry ice 5 times. After centrifugation for 10 min in a table-top microfuge (15,000× *g*, 4 °C), supernatant was collected and filtered through a 0.45 µm filter (Millipore). Free nucleic acids in the filtrates (not protected in viral capsids) were then digested using DNAse and RNAse enzymes to enrich for viral nucleic acids. Nucleic acids were then extracted (MagMAX Viral RNA Isolation Kit, Ambion, Inc, Austin, TX, USA) [7] and amplified by random RT-PCR followed by use of the Nextera™ XT Sample Preparation Kit (Illumina) to generate a library for Illumina MiSeq (2 × 250 bases) with dual barcoding as previously described (method N1) [8].

### 2.4. Bioinformatics

An in-house analysis pipeline was used to analyze sequence data that is posted in GitHub: https://github.com/xutaodeng/virushunter/. 

Database compilation: Human reference genome sequence and mRNA sequences (hg38) were concatenated. Bacterial nucleotide sequences were extracted from NCBI nt fasta file (ftp://ftp.ncbi.nlm.nih.gov/blast/db/FASTA/, February 2019) based on NCBI taxonomy (ftp://ftp.ncbi.nih.gov/pub/taxonomy, February 2019). We used taxonomy information to identify bacterial sequences from the NCBI nt database. Human and bacterial nucleotide sequences were compiled into bowtie2 (version 2.2.4) databases [9] for human and bacterial sequences subtraction. Then two databases were constructed: (1) virus BLASTx database was compiled using NCBI virus reference proteome (ftp://ftp.ncbi.nih.gov/refseq/release/viral/, February 2019) to which was added viral proteins sequences from NCBI nr fasta file (based on annotation taxonomy in Virus Kingdom); and (2) a non-redundant (NR) universal proteome database derived from NCBI’s nr was indexed using DIAMOND, where each sequences was labeled as virus or non-virus based on annotation taxonomy excluding Virus Kingdom. Repeats and low-complexity regions were masked using segmasker from blast+ suite (version 2.2.7) [10]. 

Preprocessing: Human host reads and bacterial reads were identified and removed by mapping the raw reads to human reference genome hg38 and bacterial genomes using bowtie2 in local search mode with other parameters set as default, requiring finding approximately 60bp aligned segment with at most 2 mismatches and no gaps [9]. Reads were considered duplicates if position 5 to 55 from 5 prime end were identical. One random copy of duplicates was kept. Low sequencing quality tails were trimmed using Phred quality score 20 as the threshold. Adaptor and primer sequences were trimmed using the default parameters of VecScreen using default parameters [10].

De novo assembly: We developed an ensemble strategy that integrated the sequential use of various de Bruijn graph (DBG) and overlap-layout-consensus assemblers (OLC) with a novel partitioned sub-assembly approach called EnsembleAssemble available in GitHub (https://github.com/xutaodeng/EnsembleAssembler) [11]; contigs and reads were both used for sequence similarity search.

Sequence similarity search: The assembled contigs and singlets were aligned to our viral proteome database using BLASTx (version 2.2.7) using *E*-value cutoff of <0.01. Matches to viral proteins are then aligned to our non-redundant (NR) universal proteome database using DIAMOND version 0.9.6 (10) to filter out non-viral hits that have better alignments to non-viral species. Reference sequences were annotated as virus or non-virus according to NCBI taxonomy in the DIAMOND NR database. When a read or contig was searched against this database using DIAMOND, this read was assigned to virus, or non-viruses according to the best hit to the reference sequence with lowest E-value. If its best hit is non-virus, this read is removed from final results.

Splicing analysis: For the parvovirus VP1 gene and the polyomavirus LT antigen genome potential splice donor (SD) and acceptor (SA) sites were predicted using the Human Splice Finder tool [12].

### 2.5. Generation of Full Genomes 

Nested PCR primers were designed based on metagenomics-derived sequences matching California sea lion parvovirus Hanchett and California sea lion polyomavirus 2 (Supplemental Table S1). PCR was carried out in a total volume of 25 μL. PCR mixes contained 3 μL of extracted DNA, 1.25 units Takara La Taq DNA polymerase (Takara, Mountain View, CA 94043), 10 pmol of each primer sets (Supplemental Table S1), dNTP mixture (Takara) and the supplied 10× La PCR buffer II (Takara). First round PCR products were used as template in a 25 μL nested PCR. After an initial denaturation at 94 °C for 1 min, amplification was performed for 40 cycles consisting of 20 s at 94 °C, 30 s at 55 °C, and 1.5 min at 72 °C, followed by a final extension for 10 min at 72 °C. PCR amplicons were sequenced using Sanger sequencing. 

### 2.6. Phylogenetic Analysis

The parvovirus NS1 and VP1 and polyomavirus LT antigen protein sequences were aligned using MAFFT in Geneious v10.1.3. with positions containing gaps and missing data removed. Phylogenetic trees of parvoviruses VP1 gene and polyomaviruses were constructed using the maximum likelihood method with two substitution models: Le_Gascule_2008 model (LG) with frequencies and gamma distributed, invariant sites (G + I) model MEGA software version X [13]. The aa phylogenetic trees of parvoviruses NS1 gene were constructed using the maximum likelihood method with two substitution models: General Reverse Transcriptase with frequencies and gamma distributed, invariant sites (G + I) model MEGA software version X [14]. The substitution models were selected based on the results of the Best Model search of MEGA X. The percentage of trees in which the associated taxa clustered together is shown next to the branch points. Initial tree(s) for the heuristic search were obtained by applying the neighbor-joining method to a matrix of pairwise distances estimated using the maximum composite likelihood (MCL) approach. The tree is drawn to scale, with branch lengths measured in the number of substitutions per site. For both viral genome the taxa selected for phylogenetic analyses consisted of the closest relative proteins sequences based on BLASTp, any other parvovirus or polyomavirus from California sea lions, and representative taxa from the same and different genera in the same family. Evolutionary analyses were conducted in MEGAX [15]. 

### 2.7. RNAScope^®^ In Situ Hybridization

Representative unstained histologic sections including mesenteric, stomach, gastric-associated lymph node, urinary bladder, and kidney were selected for RNAScope^®^ in situ hybridization chromogenic manual assay using the RNAScope^®^ 2.5 HD Detection Kit Red and 20-pair oligonucleotide probes targeted against the parvovirus NS1 protein (Catalog # 570561) and the polyomavirus LT proteins (Catalog # 808101) according to the manufacturer’s directions (Advanced Cell Diagnostics, Inc., Newark, CA). A negative control probe targeted against the DapB gene from the Bacillus subtilis strain SMY (Catalog # 310043) was used on all sections in parallel with the target probe. To determine adequate quality of the histologic sections, a probe developed to target the Csl GAPDH (Catalog # 570571) was used as a positive control.

## 3. Results 

### 3.1. Necropsy

**Figure 1:**
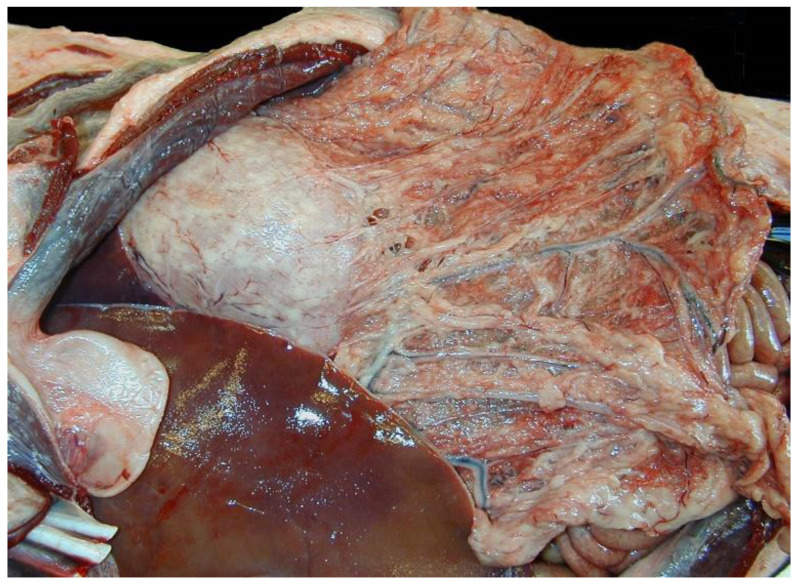
Representative California sea lion with steatitis and polyserositis, peritoneal cavity. The omental adipose is friable, tan, and reddened with congested vessels and firm nodules surrounding the arcuate vasculature. The gastric serosa is overlain by adherent white to pale tan slightly raised plaques. The liver margins are sharp and covered with a thin layer of tan friable material.

Upon gross examination, this sea lion had severe, diffuse steatitis with pleuritisperitonitis (polyserositis) characterized by abnormal white, pale tan to yellow, firm, and friable omental and mesenteric adipose tissue and roughened pleural, peritoneal, and serosal surfaces. The lungs were firm, pale tan and pink, and failed to collapse completely, consistent with interstitial pneumonia. The mediastinal and pericardial adipose were thickened, firm, pale tan and friable. as in the peritoneal cavity. The stomach and intestinal tract had multifocal mural thickening and the gastric and mesenteric lymph nodes were enlarged, firm, and tan. The kidneys, pancreas, salivary glands, and skeletal muscle had tan, firm, and coalescing foci indicative of chronic inflammation. Similar gross necropsy findings were noted in 11 other Csls stranding during along the CA coastline during the same six-month period with polyserositis and steatitis (Figure 1). 

Antemortem serum biochemical analyses revealed azotemia (increased Blood Urea Nitrogen (81; Reference Range (RR): 17–41 mg/dL; increased creatinine (1.4; RR: 0.0–1.0 mg/dL), hyperphosphatemia (7.9; RR: 4.3–6.7 mg/dL)), and hypocalcemia (8.2; RR: 8.3–9.7 mg/dL), which together indicated renal disease. In addition, serum potassium (6.4; RR: 4.1–5.1 mmol/L), creatine kinase (1988; RR: 80–158 U/L), triglycerides (246; RR: 31–179 mg/dL), and aspartate aminotransferase (150; RR: 0–87 U/L) were elevated and blood glucose (71; 87–141 mg/dL) was below normal range. These latter results were attributed to starvation and inanition with muscle breakdown and lipid mobilization. Hematology indicated a mild leukocytosis (WBC 29.7; RR: 9.4–22.8 10^3^/mm^3^) with neutrophilia.

### 3.2. Histology 

**Figure 2:**
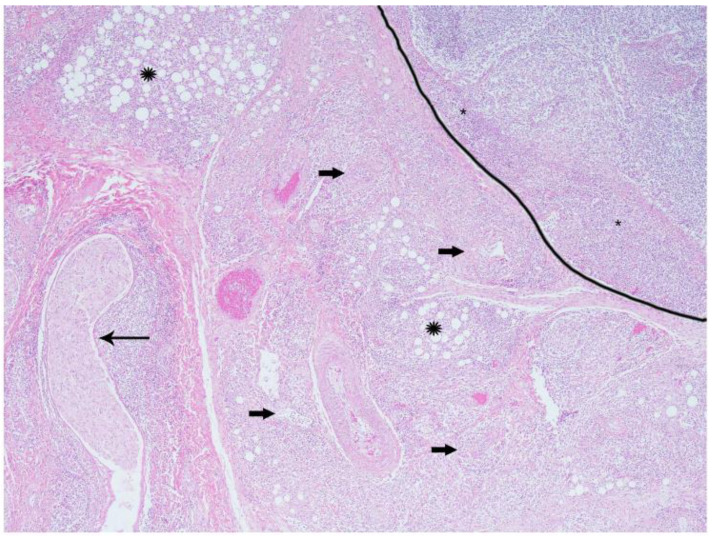
California sea lion, gastric lymph node and mesentery. The lymph node (outlined) has loss of normal cortical architecture with extensive mononuclear infiltrates in the subcapsular sinuses (asterisks). Some adjacent vessels (block arrows), nerve (arrow), and adipose tissues (starbursts) are surrounded and partially obscured by high numbers of mononuclear cells. Hematoxylin and eosin, 4× magnification.

**Figure 3:**
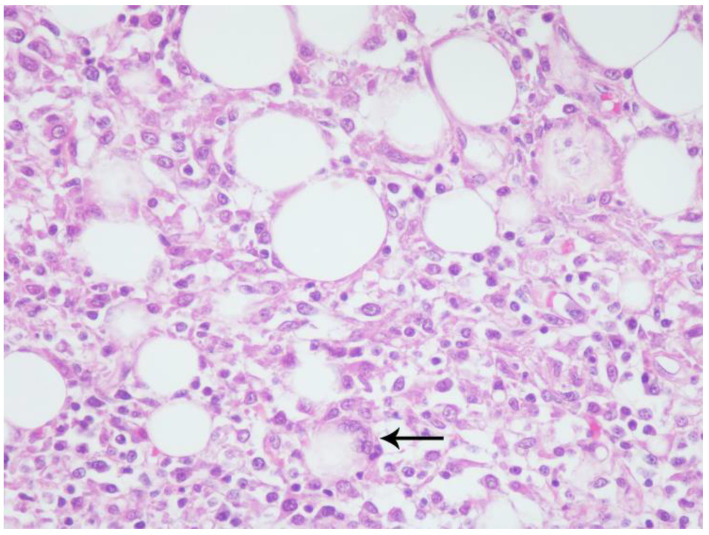
California sea lion, mesenteric adipose. Adipocytes vary in size and are surrounded, separated, and partially to completely replaced by epithelioid and foamy macrophages with low numbers of lymphocytes, plasma cells, and neutrophils. Rare adipocyte remnants are within multinucleated giant cells (arrow). Hematoxylin and eosin, 40× magnification.

**Figure 4:**
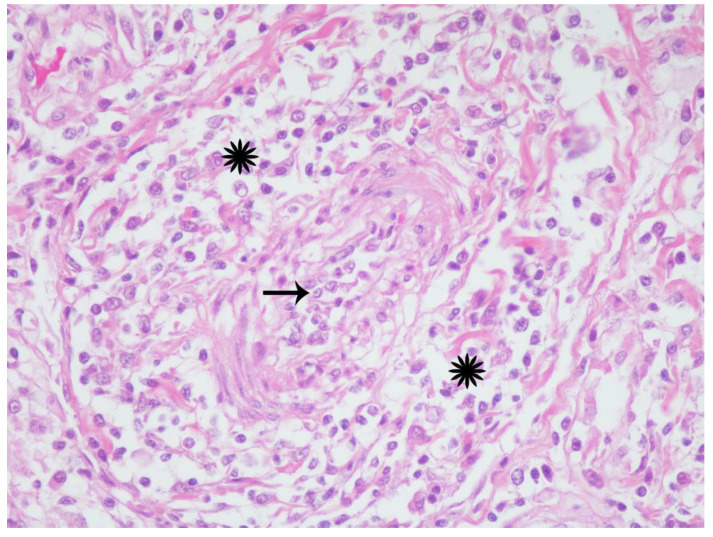
California sea lion, mesenteric arteriole. A medium caliber arteriole has circumferential mural thickening (starbursts) and luminal attenuation (arrow) with infiltration and separation of collagen fibers and smooth myocytes by similar inflammatory cells. Hematoxylin and eosin, 40× magnification.

Histologic examination confirmed chronic inflammation in the thoracic (mediastinal and pericardial) and peritoneal (mesenteric and omental) adipose tissues (steatitis); lymph nodes; and various parenchymal organs, predominantly lungs, kidneys, and the gastrointestinal tract. Affected tissues were expanded and replaced by variably dense accumulations of macrophages and mononuclear cells amongst edematous, sparsely collagenous, and highly cellular (immature) to dense collagenous (mature) fibrous connective tissue (fibrosis) (Figure 2). Macrophages were large with abundant eosinophilic foamy cytoplasm, poorly defined cell borders, and large vesicular nuclei. Intermixed with macrophages were few to numerous lymphocytes, plasma cells, and neutrophils and occasional multinucleated giant cells (Figure 3). Small to medium-caliber blood vessels including arterioles and venules, had mild to moderate mural thickening with large smudged smooth muscle cells and mononuclear cell infiltrates with reactive endothelium (Figure 4). Pulmonary parenchyma, thoracic, abdominal, and to a lesser extent, peripheral lymph nodes, had partial to nearly complete effacement of normal architecture by similar histiocytic and mononuclear infiltrates with associated fibrosis. Renal interstitium, pelvices, capsule, and peri-renal fat were similarly affected as were the serosal surfaces of the gastrointestinal tract. Skeletal and cardiac muscle had myocyte degeneration and necrosis with surrounding macrophages, fibrosis, and large regions of dystrophic mineralization. The liver, salivary glands, endocrine glands, reproductive tract with gonads, and urinary bladder were similarly though lesser affected. Representative sections stained with various special histochemical stains including Gram, Giemsa, and Gomori’s methenamine silver revealed no causative agents including fungi, bacteria, and protozoan or metazoan parasites. Macrophages within the lesions did not contain ceroid pigments based on lack of autofluorescence and negative staining with Ziehl–Neelsen and periodic acid-Schiff (PAS), further indicating absence of fungal and mycobacterial infections [16]. 

Histologic lesions were similar in the other 11 Csls though no other causative agents could be identified in any of the affected animals on routine histology, special stains, or immunohistochemistry. Vitamin E deficiency has been associated with numerous disease processes in marine mammals including muscular degeneration, steatitis, liver necrosis, vasculopathy, anemia, and impaired reproductive capacity [17]. Vitamin E levels were evaluated in livers and sera from four affected and four age-matched control Csls and results were inconclusive as there were no differences between affected or control animals and published reference ranges for this species do not exist. 

Additional presumably unrelated lesions included mild lungworm infection (*Parafilaroides decorus*) and low numbers of lymphocytes, macrophages, and plasma cells within the meninges and choroid, which were considered non-specific inflammation and incidental in a free-ranging sea lion.

### 3.3. Protein Electrophoresis, Serology, and Immunohistochemistry

Serum protein electrophoresis was completed and had an increased total protein (9.1; canine Reference Range (RR): 5.3–7.0 g/dL), α1 (acute phase protein, 0.66; canine RR: 0.1–0.31 g/dL), and gamma globulins (3.73; canine RR: 0.34–1.09 g/dL) with decreased α2 (acute phase protein, 0.7; canine RR: 0.94–1.63 g/dL) and a decreased albumin:globulin (A/G) ratio (0.44; canine RR: 0.51–1.14).

Serum had no detectable antibody titers against *Leptospira* spp. based on the microscopic agglutination test (MAT). 

Sections immunostained for feline enteric coronavirus had no evident positive staining (Supplemental Figure S1). Results were interpreted in the context of appropriately staining positive control tissues.

### 3.4. Viral Metagenomics

A frozen (−80 °C) mesenteric lymph node tissue sample was processed by homogenization, filtration, and nuclease treatment to reduce concentration of non-capsid protected nucleic acids. Viral genomes where then extracted and randomly amplified and sequenced on the Illumina MiSeq platform (250 bases paired end reads). A total number of ~2 million reads were generated available at NCBI’s Sequence Reads Archive under GenBank accession number PRJNA639577. Mammalian viruses detected with BLASTx translated protein matches (*E* score < 10^−10^) consisted of a novel parvovirus, polyomavirus, rotavirus, anellovirus, and previously described adenovirus and bocavirus from other Csls. 

### 3.5. Novel California Sea Lion Protoparvovirus

De novo assembly generated a 4620 bases contig. Because a middle region of that contig was covered by only two reads it was confirmed by nested PCR and Sanger sequencing of the amplicons (Materials and Methods). This contig corresponded to a partial genome (incomplete un-translated terminal repeats) of a novel parvovirus we named California sea lion parvovirus Hanchett with typical ORF organization encoding NS1 non-structural and VP1 structural proteins (GenBank accession MN982959). Highly conserved protein domains were identified confirming the detection of a parvovirus genome. The NS1 ATP- or GTP-binding Walker A loop motif (GxxxxGKT/S; GPASTGKS) and Walker B motif (uuuuD/ED/E; VIWIEE) plus the two rolling circle replication motifs (xuHuHuuux; TKLHTHLIL and uxxYux-Kxx; VLTYTHKQT) (x: any amino acids, u: uncharged amino acids, and -: any amino acid or none) were identified [18,19,20]. The phospholipase A2 (PLA2) catalytic residues (HD and D) and its highly conserved calcium-binding site (YLGPG) were found at the N-terminus of the VP1.

**Figure 5:**
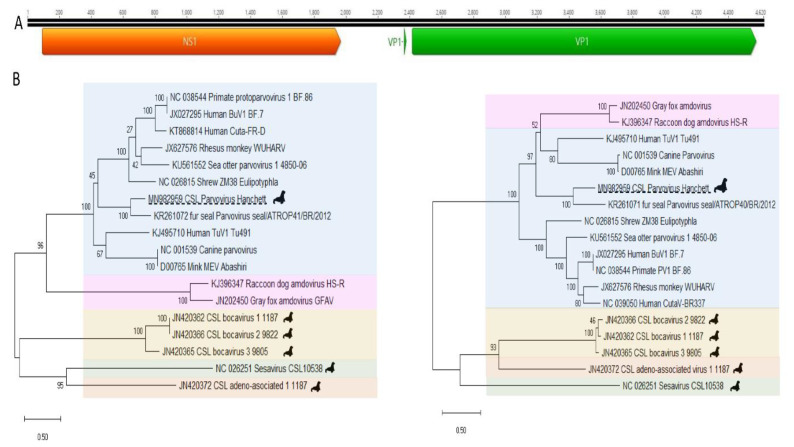
(**A**) California sea lion parvovirus Hanchett 2 ORF map and splicing motif as described in GenBank accession number MN982959. ORF structure of novel protoparvovirus. (**B**) Phylogenetic trees were constructed using the Maximum likelihood method with two substitution models: Le_Gascule_2008 model (LG) with frequencies and gamma distributed, invariant sites (G + I) model MEGA software version X. NS1 tree on left. VP1 tree on right. (Blue; protoparvovirus, purple; amdoparvovirus, yellow; bocaparvovirus, green; copiparvovirus, and orange; dependoparvovius.)

The predicted complete capsid protein (VP1) alignment showed closest identity of 57%, 40%, and 37%, to the corresponding proteins of fur seal parvovirus (KR261071), tusavirus (KJ495710) (of possible human origin), and human CutaV-BR337 (NC 039050), respectively, all in the *Protoparvovirus* genus (Figure 5). The nonstructural protein (NS1) aa alignment showed closest identity of 45%, 44%, and 40%, to the proteins of a partial fur seal parvovirus (KR261071), tusavirus (KJ495710), and mink enteritis virus strain Abashiri (D00765) also in genus *Protoparvovirus* (Supplementary Table S2). The criteria for reporting a new species within a genus of the *Parvoviridae* is that the NS1 protein must be less than 85% identical to that of a previously reported parvovirus [21]. Because the closest NS1 protein (from a fur seal) showed <85% identity this genome may belong to a new *Protoparvovirus* species pending ICTV review.

### 3.6. Novel California Sea Lion Polyomavirus

**Figure 6:**
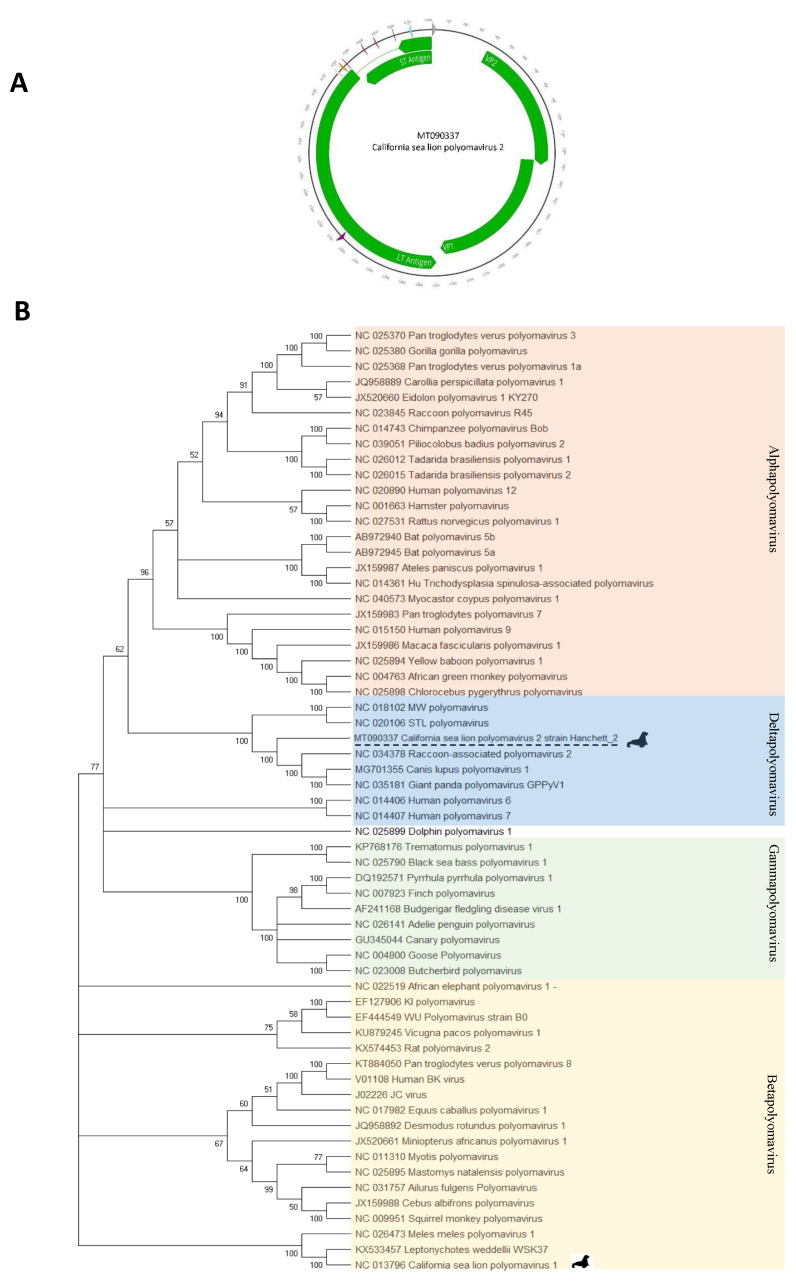
(**A**) California sea lion polyomavirus 2 ORF map and protein and splicing motifs as described in GenBank accession number MT090337. (**B**) Phylogenetic tree of LT was constructed using the maximum likelihood method with two substitution models: Le_Gascule_2008 model (LG) with frequencies and gamma distributed, invariant sites (G + I) model MEGA software version X. LT antigen genome potential splice donor (SD) and acceptor (SA) sites were predicted using the Human Splice Finder tool [12].

De novo assembly generated a 4436 bases contig. The complete DNA genome of a novel polyomavirus we called California sea lion polyomavirus 2 was completed following nested PCR and Sanger sequencing to fill a short gap leading to a complete circular 4840 bases genome (see materials and methods). That genome showed typical VP1, VP2, ST, and LT antigen ORFs organization (GenBank accession MT090337). The LT antigen contained the conserved domains HPDKGG, LXCXE, Walker A loop (G/AxxxxGKT/S; GPINSGKT), and zinc binding (CXXC-CLVC and CFSC) motifs [22] (Figure 6A) further confirming the detection of a polyomavirus.

The predicted complete LT antigen proteins alignment of California sea lion polyomavirus 2 showed the closest identity of 62%, 61%, and 60%, to the corresponding proteins of Canis lupus polyomavirus 1 (MG701355), Raccoon-associated polyomavirus 2 (NC_034378.1), and Giant panda polyomavirus (NC_035181), respectively (Supplementary Table S2).

Phylogenetic analysis indicated that the virus belonged to the deltapolyomavirus clade and was most closely related to polyomaviruses infecting other carnivores (Figure 6B). 

### 3.7. Other Viruses in Lymph Node

Sequences from another four eukaryotic viruses were also detected. Rotavirus reads and contigs, ranging in size from 243 to 786 nucleotides, were identified. Different regions (matching NSP3, NSP5, VP1, VP2, VP3, and VP4) showed closest amino acid identity to rotavirus species I genomes ranging from 52% to 79% (GenBank accession MT733332–MT733337). 

One 1664 bases contig (GenBank accession MT741090) showed best match (32% aa identity) to the ORF1 protein of Leptonychotes weddellii anellovirus genome (KY246504).

A Csl adenovirus 1 genome (GenBank accession MT610364) was partially sequenced (74% coverage of expected 31,709 bases genome) showing 99% nucleotide identity to Csl adenovirus 1 strain Zc11-030 (KJ563221) [18].

A single bocavirus read (GenBank accession MT741091) showing 89% amino acid identity to that of previously described Csl bocavirus 4 VP1 (JN420363) was also identified [23]. 

### 3.8. RNAScope^®^ In Situ Hybridization

RNAScope^®^ in situ hybridization (ISH) technology was employed in attempts to detect viral mRNA in various tissues using histologic sections of stomach, gastric-associated lymph node, mesentery, urinary bladder, and kidney. Using custom probes designed to detect viral mRNA of the parvovirus and polyomavirus, no RNA signal could be observed from the two DNA viruses tested. Thus, we conclude that the level of viral replication of these two viruses in the tissues analyzed was minimal. The negative probe (DapB) and positive probe (Csl GAPDH) were applied to replicate histologic sections of these tissues concurrently. No signal was detected in sections using the negative probe. In sections incubated with the Csl GAPDH probe, a majority of leukocytes, epithelial cells, and endothelial cells had intensely positive signal indicating quality RNA in tissue sections. 

## 4. Discussion

This emaciated sea lion with polyserositis and steatitis was one of 12 California sea lions that stranded during a 6-month period in 2010 with similar gross and histologic lesions. The clustered nature of these strandings with similar morphologic pattern of inflammatory lesions was highly suggestive of an infectious etiology, though no microorganisms could be identified through ancillary testing for bacterial, fungal, parasitic, and protozoal organisms. Using viral metagenomics, we identified multiple viruses in a single mesenteric lymph node. We sequenced the complete or near complete genome of two novel viral DNA genomes and partial genomes of another four viruses. 

A 2011 metagenomics analysis of feces from wild Csl described multiple closely related genomes from the *Bocaparvovirus* genus and one genome from the *Dependoparvovirus* genus [23]. In 2015 another parvovirus, in the *Copiparvovirus* genus, was described in a Csl suffering from malnutrition and pneumonia [24]. We describe here the genome of the first Csl protoparvovirus which was most closely related to a fur seal parvovirus. Another parvovirus, member of the *Bocaparvovirus* genus was also detected here but at lower apparent viral load in the lymph node analyzed with only a single sequence read identified. In feces of wild Csl bocaviruses were the second most common viruses detected (38% of 47 animals) after astroviruses found in 51% of these animals [23]. The closest known relative to Csl bocaviruses is the canine minute virus [25], which can cause diarrhea and respiratory problem in canine pups [26,27]. 

The first Csl polyomavirus genome (Csl polyomavirus 1) was described in a glossal fibropapilloma [28] and subsequently reported in another Csl also with a proliferative tongue lesion [26]. The Csl polyomavirus 2 sequenced here showed typical genome architecture and its closest, although still divergent, relative was Canis lupus polyomavirus 1 (MG701355), possibly reflecting an ancient common ancestor and possible cross species transmission between these two carnivores. The Csl analyzed here did not display any visible tumors and there was no evidence of proliferative disease histologically. A possible carcinogenic potential for Csl polyomavirus 2, as seem possible for Csl polyomavirus 1 [25,26], therefore remains unsupported although it should be noted that most natural infections with carcinogenic polyomavirus remain asymptomatic [29,30]. A partial rotavirus genome showed significant divergence from its closest known relative in species I detected in a prior metagenomics analysis of Csl feces [23]. Diverse rotaviruses from species I are therefore replicating in wild Csl. A large fraction of a previously described adenovirus genome was also sequenced. This virus, initially observed by electron microscopy in a Csl with hepatomegaly [31], has been associated with hepatitis in wild Csl [32] as well as in South American sea lion (*Otaria flavescens*) and South African fur seal during an aquarium outbreak [33]. Csl adenovirus 1 was also detected in another captive Hawaiian monk seal (*Neomonachus schauinslandi*) with anorexia and renal disease [34]. The closest relative is a genome from a Chinese derived porcine mastadenovirus showing 54% nucleotide identity [35]. Sequence reads from anelloviruses were also detected. Anelloviruses are common among animals and generally considered commensal infections [36,37] A prior study has reported anelloviruses in wild Csl [24].

The virome in the mesenteric lymph node from this Csl therefore consisted of a variety of mammalian viruses some of which alone or in combination could conceivably be involved in this disease process. In situ hybridization for RNA expression of the two viruses that yielded the most complete genomes during the metagenomics analysis (both DNA viruses) yielded negative results in the tissues analyzed indicating that the sites of viral replication were in other tissues. Alternatively the presence of viral genomes in the mesenteric lymph node from which they were sequenced may reflect depots of non-replicating viral particles persisting for antigenic stimulation. 

It therefore remains to be determined which, if any, of these viruses are causally linked to this animal’s disease. Analysis of other Csl’s with similar lesions will be required to determine possible association with the viruses described here and this unusual and rare syndrome. Unfortunately, additional samples from other affected sea lions were not available for metagenomic analysis. Viral metagenomics could prove useful for pathogen detection in future free-ranging pinniped cases which present with similar inflammatory syndromes of unknown origin. 

## Supplementary Materials

The following are available online at https://www.mdpi.com/1999-4915/12/8/793/s1, Figure S1. Lymph node vessel FIP IHC 20x. Table S1. Primers used to complete genomes. Table S2. Parvovirus NS1, VP1 and Polyomavirus LT antigen distance table.

## References

[1] Engelhardt F.R., Geraci J.R. (1978). Effects of experimental vitamin E deprivation in the harp seal, *Phoca groenlandica*. Can. J. Zool..

[2] Gulland F.M. (1999). Stranded seals: Important sentinels. J. Am. Vet. Med. Assoc..

[3] Addie D., Belák S., Boucraut-Baralon C., Egberink H., Frymus T., Gruffydd-Jones T., Hartmann K., Hosie M.J., Lloret A., Lutz H. (2009). Feline infectious peritonitis. ABCD guidelines on prevention and management. J. Feline Med. Surg..

[4] Garner M.M., Ramsell K., Morera N., Juan-Sallés C., Jiménez J., Ardiaca M., Montesinos A., Teifke J., Löhr C.V., Evermann J.F. (2008). Clinicopathologic features of a systemic coronavirus-associated disease resembling feline infectious peritonitis in the domestic ferret (*Mustela putorius*). Vet. Pathol..

[5] Frances M.D., Gulland L.A.D., Whitman K.L. (2018). CRC Handbook of Marine Mammal. Medicine.

[6] Colagross-Schouten A.M., Mazet J.A.K., Gulland F.M.D., Miller M.A., Hietala S. (2002). Diagnosis and seroprevalence of leptospirosis in California sea lions from coastal California. J. Wildl. Dis..

[7] Victoria J.G., Kapoor A., Li L., Blinkova O., Slikas B., Wang C., Naeem A., Zaidi S., Delwart E. (2009). Metagenomic analyses of viruses in stool samples from children with acute flaccid paralysis. J. Virol..

[8] Li L., Deng X., Mee E.T., Collot-Teixeira S., Anderson R., Schepelmann S., Minor P.D., Delwart E. (2015). Comparing viral metagenomics methods using a highly multiplexed human viral pathogens reagent. J. Virol. Methods.

[9] Langmead B., Salzberg S.L. (2012). Fast gapped-read alignment with Bowtie 2. Nat. Methods.

[10] Ye J., McGinnis S., Madden T.L. (2006). BLAST: Improvements for better sequence analysis. Nucleic Acids Res..

[11] Deng X., Naccache S.N., Ng T.F.F., Federman S., Li L., Chiu C.Y., Delwart E.L. (2015). An ensemble strategy that significantly improves de novo assembly of microbial genomes from metagenomic next-generation sequencing data. Nucleic Acids Res..

[12] Desmet F.O., Hamroun D., Lalande M., Collod-Béroud G., Claustres M., Béroud C. (2009). Human Splicing Finder: An online bioinformatics tool to predict splicing signals. Nucleic Acids Res..

[13] Le S.Q., Gascuel O. (2008). An improved general amino acid replacement matrix. Mol. Biol. Evol..

[14] Dimmic M.W., Rest J.S., Mindell D.P., Goldstein R.A. (2002). rtREV: An amino acid substitution matrix for inference of retrovirus and reverse transcriptase phylogeny. J. Mol. Evol..

[15] Kumar S., Stecher G., Li M., Knyaz C., Tamura K. (2018). MEGA X: Molecular Evolutionary Genetics Analysis across Computing Platforms. Mol. Biol. Evol..

[16] Soto S., Fondevila D., González B., Gómez-Campos E., Domingo M. (2010). Multifocal granulomatous panniculitis with ceroid pigment in two Mediterranean striped dolphins (*Stenella coeruleoalba*). J. Wildl. Dis..

[17] Citino S.B., Montali R.J., Bush M., Phillips L.G. (1985). Nutritional myopathy in a captive California sea lion. J. Am. Vet. Med. Assoc..

[18] Ding C., Urabe M., Bergoin M., Kotin R.M. (2002). Biochemical characterization of *Junonia coenia* densovirus nonstructural protein NS-1. J. Virol..

[19] Fryer J.F., Delwart E., Bernardin F., Tuke P.W., Lukashov V.V., Baylis S.A. (2007). Analysis of two human parvovirus PARV4 genotypes identified in human plasma for fractionation. J. Gen. Virol..

[20] Ilyina T.V., Koonin E.V. (1992). Conserved sequence motifs in the initiator proteins for rolling circle DNA replication encoded by diverse replicons from eubacteria, eucaryotes and archaebacteria. Nucleic Acids Res..

[21] Pénzes J.J., Söderlund-Venermo M., Canuti M., Eis-Hübinger A.M., Hughes J., Cotmore S.F., Harrach B. (2020). Reorganizing the family *Parvoviridae*: A revised taxonomy independent of the canonical approach based on host association. Arch. Virol..

[22] An P., Saenz Robles M.T., Pipas J.M. (2012). Large T antigens of polyomaviruses: Amazing molecular machines. Annu. Rev. Microbiol..

[23] Li L., Shan T., Wang C., Côté C., Kolman J., Onions D., Gulland F.M.D., Delwart E. (2011). The fecal viral flora of California sea lions. J. Virol..

[24] Phan T.G., Gulland F., Simeone C., Deng X., Delwart E. (2015). Sesavirus: Prototype of a new parvovirus genus in feces of a sea lion. Virus Genes.

[25] Schwartz D., Green B., Carmichael L.E., Parrish C.R. (2002). The canine minute virus (minute virus of canines) is a distinct parvovirus that is most similar to bovine parvovirus. Virology.

[26] Carmichael L.E., Schlafer D.H., Hashimoto A. (1994). Minute virus of canines (MVC, canine parvovirus type-1): Pathogenicity for pups and seroprevalence estimate. J. Vet. Diagn. Invest..

[27] Harrison L.R., Styer E.L., Pursell A.R., Carmichael L.E., Nietfeld J.C. (1992). Fatal disease in nursing puppies associated with minute virus of canines. J. Vet. Diagn. Invest..

[28] Simpson J.T., Wong K., Jackman S., Schein J.E., Jones S.J.M., Birol I. (2009). ABySS: A parallel assembler for short read sequence data. Genome Res..

[29] Qi D., Shan T., Liu Z., Deng X., Zhang Z., Bi W., Owens J.R., Feng F., Zheng L., Huang F. (2017). A novel polyomavirus from the nasal cavity of a giant panda (*Ailuropoda melanoleuca*). Virol. J..

[30] Prado J.C.M., Monezi T.A., Amorim A.T., Lino V., Paladino A., Boccardo E. (2018). Human polyomaviruses and cancer: An overview. Clinics.

[31] Dierauf L.A., Lowenstine L.J., Jerome C. (1981). Viral hepatitis (adenovirus) in a California sea lion. J. Am. Vet. Med. Assoc..

[32] Prado J.C.M., Monezi T.A., Amorim A.T., Lino V., Paladino A., Boccardo E. (2011). Isolation of a novel adenovirus from California sea lions *Zalophus californianus*. Dis. Aquat. Organ..

[33] Inoshima Y., Murakami T., Ishiguro N., Hasegawa K., Kasamatsu M. (2013). An outbreak of lethal adenovirus infection among different otariid species. Vet. Microbiol..

[34] Cortés-Hinojosa G., Doescher B., Kinsel M., Lednicky J., Loeb J., Waltzek T., Wellehan J.F. (2016). Coinfection of California Sea Lion Adenovirus 1 and a Novel Polyomavirus in a Hawaiian Monk Seal (*Neomonachus Schauinslandi*). J. Zoo Wildl. Med..

[35] Liu S.-J., Wang Q., Li T.-T., Zhang S.-H., Li J.-Y., Wu L.-J., Qiu Y., Ge X.-Y. (2020). Characterization of the First Genome of Porcine mastadenovirus B (HNU1 Strain) and Implications on Its Lymphoid and Special Origin. Virol. Sin..

[36] Manzin A., Mallus F., Macera L., Maggi F., Blois S. (2015). Global impact of Torque teno virus infection in wild and domesticated animals. J. Infect. Dev. Ctries..

[37] Okamoto H. (2009). TT viruses in animals. Curr. Top. Microbiol. Immunol..

