# Supplementary material for: Complex Virome in a Mesenteric Lymph Node from a Californian Sea Lion (Zalophus californianus) with Polyserositis and Steatitis
Source: Viruses. 2020 Jul 23;12(8):793. doi: 10.3390/v12080793 (PMC7472147; doi:10.3390/v12080793)

Primer ID	Sequence	Length
Hanchett_Parvovirus_1,737 F1	CTGGATGGAAACCCATGGCT	1682 bp
Hanchett_Parvovirus_3,418 R1	TGTGGCGTGTAAGGGAGTTC	
Hanchett_Parvovirus_1,930 F2	GAAGATCCACTAGAGGTCGC	1250 bp
Hanchett_Parvovirus_3179 R2	CCAGGGTGTTTGGCATTGTG	
Hanchett_Polyoma_3,196 F1	GGAAAGCCATGCAACACCAG	1756 bp
Hanchett_Polyoma_84 R1	CTGCCTGCCTCTTACCCTTT	
Hanchett_Polyoma_3,596 F2	CCTGAGGATGGGCTCTCTCT	1029 bp
Hanchett_Polyoma_4654 R2	AACCCCACTCCCACCTATGA	

Supp Table 1. PCR primers used for nPCR to complete parvovirus and polyomavirus genomes. F1/R1 correspond to first PCR round while F2/R2 correspond to second round PCR.

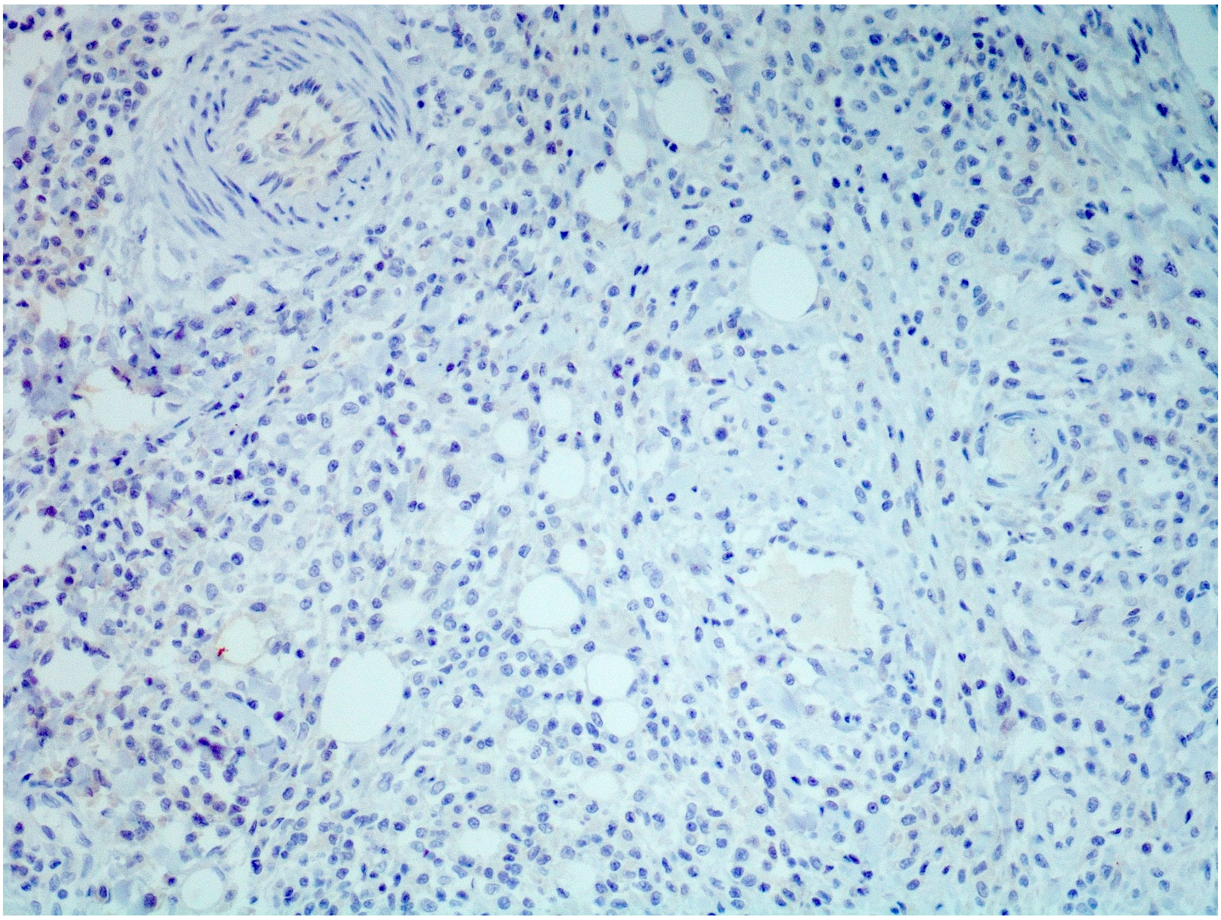

Supplement: Supplementary file 1 [file viruses-12-00793-s001.zip › Supp table 1.pdf]
